# Effectiveness of School-Based Integrated Mental Health and Substance Use Prevention Programs for Adolescents: A Systematic Review and Meta-Analysis

**DOI:** 10.3390/bs16091498

**Published:** 2026-08-26

**Authors:** Jihye Shin, Kyung-Young Hong

**Affiliations:** College of Nursing, Eulji University, Uijeongbu 34824, Republic of Korea; jhshin@cup.ac.kr

**Keywords:** adolescent, school-based prevention, substance use prevention, mental health promotion, integrated prevention, resilience, binge drinking, eHealth, systematic review, meta-analysis

## Abstract

School-based prevention programs increasingly incorporate mental health-related components to prevent substance use among adolescents. This study aimed to evaluate the effectiveness of integrated school-based mental health and substance use prevention programs for adolescents through a systematic review and meta-analysis. A systematic literature search was conducted across 12 electronic databases for studies published between January 2016 and April 2026. Randomized controlled trials (RCTs) and cluster randomized controlled trials evaluating school-based integrated prevention programs for adolescents were included. Random-effects meta-analyses were performed for substance-use outcomes, including alcohol-related outcomes and binge drinking, using pooled odds ratios (ORs) and 95% confidence intervals (CIs). Mental health and psychosocial outcomes were synthesized narratively because heterogeneity in outcome definitions, measurement instruments, and assessment periods precluded meaningful quantitative pooling. A total of 16 reports representing 10 independent trials were included in the systematic review, and five independent trial comparisons were included in the primary meta-analysis. Integrated prevention programs significantly reduced alcohol-related behaviors among adolescents compared to control groups (OR = 0.56, 95% CI = 0.37–0.86, *p* = 0.008). Significant preventive effects were also observed for binge drinking outcomes (OR = 0.32, 95% CI = 0.16–0.64, *p* = 0.001). Most interventions incorporated emotional regulation, resilience enhancement, coping skills, stress management, and help-seeking promotion. Digital and online approaches were frequently used. Narrative findings suggested potential benefits for selected psychosocial and mental health-related outcomes; however, these outcomes were not quantitatively pooled and were not consistently sustained across follow-up periods. Integrated school-based prevention programs incorporating mental health-related components demonstrated beneficial effects on alcohol-related behaviors and binge drinking among adolescents. Evidence regarding mental health and psychosocial outcomes was based on narrative synthesis and remains less conclusive. These findings support the importance of integrated prevention approaches that simultaneously address substance use and mental health in school settings. However, additional large-scale RCTs with standardized outcome measures and long-term follow-up are needed to strengthen the current evidence base.

## 1. Introduction

Adolescence is a critical developmental period characterized by rapid physical, emotional, and social changes during which various health-risk behaviors emerge. Substance use, including alcohol, tobacco, cannabis, and other drugs, is often initiated during adolescence, and early use has been associated with increased risks of substance dependence and mental health problems in adulthood (Teesson et al., 2020). Adolescent substance use is closely linked to depression, anxiety, impulsivity, academic difficulties, interpersonal problems, and increased suicide risk, potentially impairing long-term psychosocial functioning (Newton et al., 2022b; Margherio et al., 2025). Furthermore, repeated substance use during adolescence may negatively affect brain development and emotional regulation, emphasizing the importance of early preventive interventions (Butzer et al., 2017; Menezes et al., 2025).

Recent evidence suggests that adolescent substance use is influenced by multiple psychosocial factors, including peer relationships, family environment, school context, stress, and emotional regulation abilities (Garcia-Cerde et al., 2023; Beckman et al., 2017; Edalati et al., 2019). Accordingly, prevention strategies have evolved beyond simple drug education toward strengthening psychosocial protective factors such as stress management, decision-making skills, emotional regulation, social competence, and resilience (Kulis et al., 2017, 2025).

Schools are considered ideal settings for substance use prevention because they provide access to large populations of adolescents and allow universal prevention approaches to be implemented efficiently (Hodder et al., 2017). School environments also facilitate the integration of prevention programs into adolescents’ daily lives through peer interactions and teacher support systems (Gabrhelik et al., 2012; Kulis et al., 2019).

In recent years, school-based prevention programs have increasingly incorporated mental health-related components, reflecting recognition of the close relationship between substance use and mental health problems. Depression, anxiety, and stress have been identified as important risk factors for adolescent substance use, leading to greater integration of substance use prevention and mental health promotion (Teesson et al., 2020; Halladay et al., 2024). For example, the Climate Schools–Combined program demonstrated positive effects on both substance-related outcomes and mental health symptoms, with some benefits persisting into early adulthood (Teesson et al., 2020, 2024). These findings support integrated prevention approaches that simultaneously address substance use and mental health.

The delivery of prevention programs has also changed considerably with advances in digital technology. While traditional programs primarily relied on face-to-face instruction, recent interventions increasingly utilize web-based, mobile-based, and application-based approaches (Champion et al., 2023). Digital programs such as Climate Schools, SmartCoach, and Mind Your Mate have reported positive outcomes related to substance use, stress reduction, mental health promotion, and help-seeking behaviors (Champion et al., 2016, 2018; Haug et al., 2021; Birrell et al., 2023). These interventions offer advantages in accessibility, scalability, and standardized delivery.

At the same time, prevention strategies have diversified. In addition to universal prevention, selective and combined approaches have been developed. Programs such as Preventure demonstrated effectiveness through personality-targeted selective prevention (Debenham et al., 2021; Lammers et al., 2017), while the Climate and Preventure (CAP) study integrated universal and selective approaches and reported long-term preventive benefits for certain outcomes (Newton et al., 2018; Teesson et al., 2017). Furthermore, recent programs increasingly emphasize professional help-seeking and psychosocial competence. For example, MAKINGtheLINK promoted help-seeking for mental health and substance use concerns (Lubman et al., 2020), whereas #Tamojunto2.0 highlighted the importance of peer influence and alcohol-related beliefs in prevention efforts (Garcia-Cerde et al., 2023).

Despite the growing body of research, considerable heterogeneity exists across intervention types, delivery methods, program components, and outcome measures. In particular, although digital-based interventions and integrated mental health approaches have increased substantially, comprehensive syntheses of their characteristics and effectiveness remain limited. Previous reviews have often focused on specific substances or individual prevention strategies, making it difficult to understand the broader effectiveness of school-based programs integrating mental health-related components.

Therefore, this systematic review and meta-analysis examined the characteristics, delivery methods, prevention strategies, and outcomes of school-based programs integrating substance-use prevention with mental health-related components for adolescents. Quantitative synthesis was limited to substance-use outcomes for which sufficiently comparable data were available, whereas mental health and psychosocial outcomes were synthesized narratively because of heterogeneity in their definitions, measurement instruments, and assessment periods. The findings are expected to inform the development and implementation of integrated school-based prevention programs while clearly distinguishing evidence for substance-use outcomes from the less conclusive evidence concerning mental health outcomes.

## 2. Methods

This study was conducted as a systematic review and meta-analysis to comprehensively examine the characteristics and effectiveness of school-based substance use prevention programs for adolescents. This study was conducted in accordance with the PRISMA 2020 guidelines (Supplementary Table S1) and was prospectively registered in PROSPERO (ID: CRD420261374182).

In addition, the study was conducted according to a predefined protocol, and exploratory analyses and sensitivity analyses were performed to further investigate heterogeneity identified in the primary analyses.

### 2.1. Search Strategy

Electronic database searches were conducted in April 2026 using PubMed, Embase, Cochrane Library, CINAHL, KMbase, KoreaMed, PsycINFO, ScienceON, DBpia, KISS, RISS, and PQDT to identify studies related to school-based substance use prevention programs for adolescents. Studies published between January 2016 and April 2026 were considered for inclusion. The search period beginning in 2016 was selected because school-based preventive interventions integrating substance use prevention and mental health-related components, particularly digital-based and resilience-oriented approaches, began to be more systematically developed and increasingly evaluated through randomized controlled trials during this period.

The search strategy was developed based on four key concepts: adolescents, school-based settings, substance use, and mental health-related prevention. To maximize search comprehensiveness, both Medical Subject Headings (MeSH) terms and free-text terms were used. The search terms included combinations of keywords such as “adolescent,” “teen,” “youth,” “school-based,” “school program,” “substance use,” “alcohol,” “smoking,” “cannabis,” “mental health,” “depression,” “anxiety,” “stress,” “resilience,” “well-being,” “prevention,” and “intervention.” Boolean operators (“AND” and “OR”) were used to combine search terms appropriately. The detailed PubMed search strategy is presented in Supplementary Table S1.

To enhance search comprehensiveness, additional hand-searching of reference lists from relevant review articles and included studies was conducted. Gray literature and clinical trial registries were also reviewed, when necessary, to identify ongoing or unpublished studies.

The initial screening process was independently conducted by two reviewers (K Hong and J Shin) through title and abstract screening. Subsequently, full-text reviews of potentially eligible studies were independently performed according to predefined inclusion and exclusion criteria. Any disagreements arising during the study selection and eligibility assessment processes were resolved through discussion and consensus.

### 2.2. Study Selection Criteria

Studies were selected according to the following inclusion criteria:

(1) Participants were adolescents aged 13–18 years;
(2) Interventions were conducted in school settings and aimed to prevent substance use or reduce substance use-related risk behaviors;
(3) Interventions included mental health-related components such as stress management, emotional regulation, resilience, or help-seeking behaviors;
(4) Comparison groups included no intervention, wait-list controls, general education, usual care, or active control conditions;
(5) Outcomes included substance use, mental health, psychosocial variables, or help-seeking behaviors measured using validated instruments; and
(6) Study designs were randomized controlled trials (RCTs) or cluster randomized controlled trials (cluster RCTs).

Studies were excluded if they involved college students or adults, focused on treatment rather than prevention, did not include mental health-related components, did not report substance use-related outcomes, or did not use randomized controlled designs (Galán et al., 2023). In addition, non-peer-reviewed literature such as conference abstracts, editorials, review articles, and dissertations, as well as studies for which full texts were unavailable, were excluded. After removing duplicate records, two reviewers independently conducted a two-stage screening process consisting of title and abstract screening followed by full-text review. Any disagreements arising during the study selection process were resolved through discussion and consensus. The overall selection process was conducted in accordance with the PRISMA 2020 guidelines.

### 2.3. Data Extraction

Data extraction was independently performed by two reviewers using a standardized data extraction form. The following information was extracted:

(1) Participant and setting characteristics, including sample size, mean age, sex distribution, number and type of participating schools, geographical or rural–urban setting, and racial, ethnic, cultural, and socioeconomic characteristics, when reported;
(2) Intervention characteristics, including intervention name, intervention type, mental health-related components, delivery methods, number of sessions, intervention duration, and intervention providers;
(3) Control group characteristics, including wait-list controls, general education, no-intervention controls, and active control conditions; and
(4) Outcome variables related to substance use, mental health, psychosocial variables, and help-seeking behaviors.

Information that was not provided in the original studies was recorded as “not reported” and was not inferred by the reviewers. For studies included in the meta-analysis, statistical data such as event data, means, standard deviations (SDs), sample sizes, odds ratios (ORs), and confidence intervals (CIs) were extracted whenever available (Teesson et al., 2020). When studies reported statistical values such as t values, F values, confidence intervals, or *p* values, these statistics were used to calculate effect sizes according to established meta-analytic procedures (Moore et al., 2020). Studies that did not provide sufficient statistical information were excluded from quantitative synthesis to maintain methodological rigor. Any discrepancies arising during the data extraction process were resolved through discussion and consensus between the reviewers.

Multiple publications arising from the same parent trial were identified by comparing trial registration numbers, sample sizes, participating schools, intervention groups, recruitment periods, and participant characteristics. These publications were linked at the parent–trial level during data extraction.

### 2.4. Interventions and Outcome Measures

The interventions included in this review consisted of school-based prevention programs aimed at reducing adolescent substance use and promoting mental health. The programs were based on various approaches, including resilience-oriented approaches, social influence approaches, life-skills training, psychosocial interventions, and digital-based preventive interventions (Hodder et al., 2017; Kulis et al., 2017; Griffin et al., 2026). Although intervention methods varied across studies, most programs commonly aimed to reduce substance use-related risk behaviors while enhancing emotional regulation, coping skills, decision-making abilities, stress management, resilience, and help-seeking behaviors. The primary outcomes of this study included alcohol use, smoking, cannabis use, binge drinking, and other substance use-related outcomes (Teesson et al., 2020; Gardner et al., 2025). Secondary outcomes included depression, anxiety, stress, resilience, emotional regulation, psychosocial functioning, and help-seeking behaviors reported in the included studies (Newton et al., 2020; Lawler et al., 2024). Outcome variables were measured using validated self-report instruments or standardized assessment tools reported in the included studies (Margherio et al., 2025; Kulis et al., 2025).

### 2.5. Quality Assessment

The methodological quality of the included studies was assessed using the Cochrane Risk of Bias 2 tool for cluster randomized trials (RoB 2 for Cluster RCTs). Two reviewers independently evaluated the following domains: randomization process, deviations from intended interventions, missing outcome data, measurement of outcomes, and selection of reported results. Each study was classified as having “low risk,” “some concerns,” or “high risk” according to the RoB 2 guidelines. Any disagreements arising during the quality assessment process were resolved through discussion and consensus.

### 2.6. Statistical Analysis

Meta-analyses were conducted using RevMan version 5.4 (The Cochrane Collaboration, Copenhagen, Denmark). Primary outcome variables were analyzed using odds ratios (ORs) and 95% confidence intervals (CIs) based on binary outcome data. For cluster randomized controlled trials (cluster RCTs), the Generic Inverse Variance (GIV) method was applied, and when necessary, log ORs and standard errors (SEs) were calculated from reported ORs and 95% CIs.

Whenever available, statistical data including event data, sample sizes, ORs, and 95% CIs were extracted for the meta-analysis (Teesson et al., 2020). When multiple publications reported outcomes from the same parent trial, they were treated as a single study source for each meta-analysis. For overlapping outcomes and assessment periods, data from the most complete or latest report were used, whereas earlier reports were retained only when they provided unique outcomes or time points. No participant sample was included more than once within the same pooled analysis.

Statistical heterogeneity was assessed using the I^2^ statistic. Because heterogeneity related to intervention characteristics, participant populations, outcome measurements, and study designs was anticipated, all analyses were conducted using random-effects models. When substantial heterogeneity was identified, potential sources were examined qualitatively by comparing substance and outcome definitions, intervention components, delivery formats, control conditions, participant characteristics, and follow-up durations across studies. Formal subgroup analysis or meta-regression was not conducted when fewer than 10 studies were available because such analyses would have been underpowered and potentially misleading. Exploratory and sensitivity analyses were additionally performed for selected outcomes when clinically and methodologically appropriate (Newton et al., 2018; Teesson et al., 2024).

Secondary cognitive and psychosocial outcomes were synthesized narratively because of substantial variation in their definitions and measurement instruments. These outcomes were based on study-specific measures and between-group changes over time; no composite psychosocial protective-factor score was calculated in this review.

Formal assessments of publication bias using funnel plots or regression-based tests, such as Egger’s test, were not performed because the primary meta-analysis included only five independent trial comparisons and the other pooled analyses included fewer studies. With fewer than 10 independent studies, these methods would have insufficient power to reliably distinguish publication bias from chance or between-study heterogeneity. Instead, selective reporting risk was considered qualitatively based on the RoB 2 domain concerning selection of the reported results, together with the availability and completeness of the outcomes reported in the included studies.

## 3. Results

### 3.1. Study Selection

**Figure 1:**
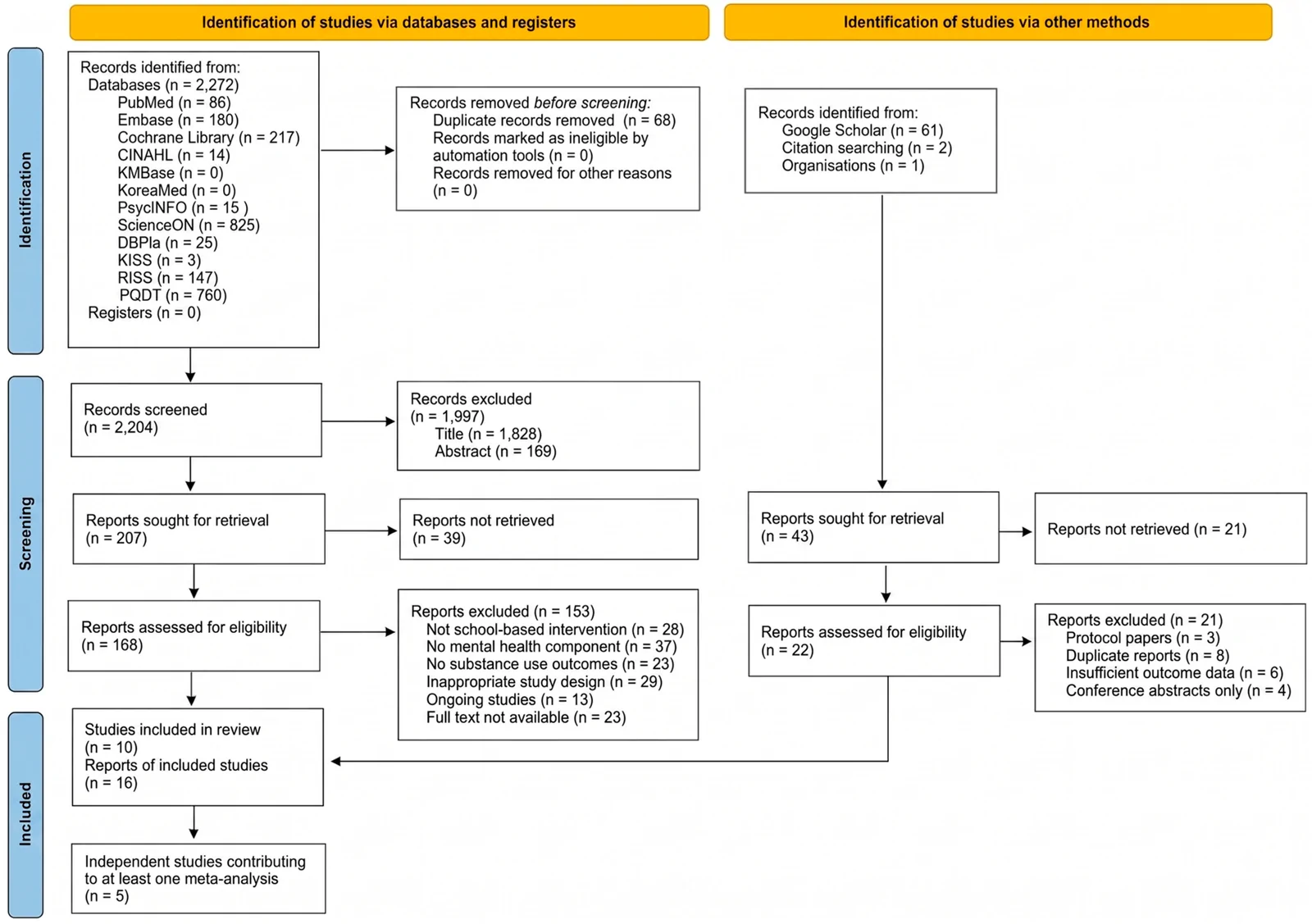
PRISMA flow diagram of study selection.

Figure 1 presents the PRISMA flow diagram of the study selection process. A total of 2272 records were identified through database searching, and additional records were identified through reference list reviews and hand-searching. After removing duplicate records, 2204 studies underwent title and abstract screening. Subsequently, 207 reports were sought for retrieval, of which 168 were assessed for eligibility. In addition, 22 reports identified through other methods were assessed for eligibility. Following full-text assessment, 16 reports representing 10 independent parent trials met all inclusion criteria and were included in the systematic review. Following full-text review, 16 reports representing 10 independent parent trials met all inclusion criteria and were included in the systematic review. Five independent trial comparisons provided sufficient quantitative data for inclusion in the primary meta-analysis.

Studies were excluded if they were not school-based interventions, did not include mental health-related components, did not report substance use-related outcomes, did not use randomized controlled designs, or if full texts were unavailable (Galán et al., 2023). The entire study selection process was independently conducted by two reviewers (K Hong and J Shin) in accordance with the PRISMA 2020 guidelines.

### 3.2. Study Characteristics

Table 1 presents the key characteristics of the included studies. Additional details regarding intervention duration, providers, control conditions, participant diversity, and school and community contexts are provided in Supplementary Table S3. A total of 16 reports derived from 10 independent randomized controlled trials or cluster randomized controlled trials were included in this systematic review. Six reports were follow-up or secondary analyses of previously reported parent trials. Champion et al. (2016) and Champion et al. (2018) reported outcomes from the same Climate Schools: Ecstasy and Emerging Drugs trial, with the latter extending follow-up to 24 months and examining implementation dose. Teesson et al. (2017), Newton et al. (2018), Debenham et al. (2021), and Newton et al. (2022b) originated from the CAP trial but examined different substance-related outcomes or follow-up periods. Similarly, Teesson et al. (2020), Newton et al. (2022a), and Teesson et al. (2024) were based on the Climate Schools Combined trial and reported different intervention comparisons or follow-up periods. These related publications were retained because they contributed distinct outcomes or extended follow-up data; however, they were not treated as independent samples within the same meta-analysis. Their parent–trial relationships are detailed in Supplementary Table S3. Most studies were conducted in Australia, while several studies were conducted in Switzerland, Brazil, and the United States.

Participants consisted of adolescents aged 13–18 years. The reported mean ages ranged from 13.2 to 15.9 years across studies. Sample sizes varied from 166 to 6386 participants.

The included studies varied considerably in the number, type, and geographical distribution of participating schools. Several Australian studies included public, independent, and Catholic schools across multiple states, whereas other studies were conducted within more geographically restricted metropolitan settings. The Swiss study reported participants’ immigration backgrounds, the Brazilian study reported socioeconomic categories, and the US study provided detailed racial and ethnic characteristics. However, race or ethnicity, socioeconomic background, and rural–urban setting were inconsistently reported across the included studies. Detailed population and contextual characteristics are presented in Supplementary Table S3.

The included interventions demonstrated substantial variability in terms of intervention approaches and delivery methods. Most studies employed universal school-based prevention programs, whereas some studies included selective prevention or integrated prevention approaches. Interventions generally integrated substance use prevention with mental health-related components such as emotional regulation, resilience, stress management, coping skills, social competence, life-skills training, and help-seeking promotion.

Regarding delivery methods, online and digital-based approaches, including web-based programs, mobile applications, and blended or hybrid formats, were frequently used. Some studies additionally incorporated face-to-face sessions, workshops, and teacher-led classroom activities. Several studies also included long-term follow-up assessments.

Primary outcome variables included alcohol use, smoking, cannabis use, binge drinking, and other substance use-related behaviors. Secondary outcomes included depression, anxiety, stress, resilience, psychosocial functioning, and help-seeking behaviors. Most studies used validated self-report instruments to evaluate intervention effectiveness.

### 3.3. Meta-Analysis of School-Based Integrated Prevention Programs

#### 3.3.1. Alcohol Use and Alcohol-Related Outcomes

The meta-analysis of alcohol-related outcomes included four studies (Teesson et al., 2020; Haug et al., 2021; Debenham et al., 2022; Slade et al., 2023). Using a random-effects model, school-based integrated prevention programs were found to significantly reduce alcohol-related behaviors among adolescents compared with control groups (OR = 0.56, 95% CI [0.37, 0.86], *p* = 0.008).

**Figure 2:**
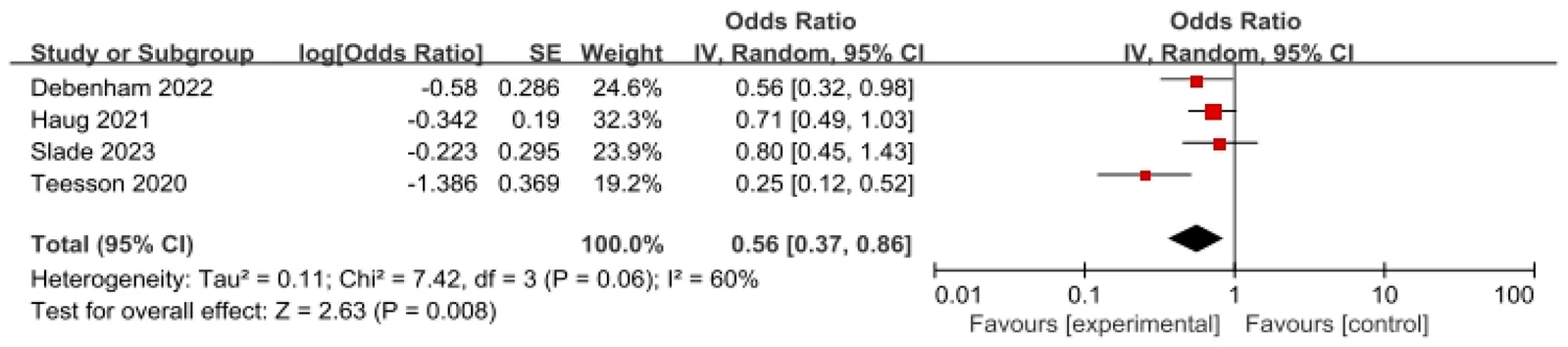
Forest plot of alcohol-related outcomes (Teesson et al., 2020; Haug et al., 2021; Debenham et al., 2022; Slade et al., 2023).

Substantial heterogeneity was observed across studies (I^2^ = 60%). Qualitative examination identified several plausible sources. First, the studies assessed different alcohol-related constructs, including alcohol initiation, any alcohol use, frequency or quantity of consumption, and high-risk drinking. Second, intervention content and delivery differed: Teesson et al. (2020) evaluated a teacher-facilitated online universal program integrating substance use and mental health content; Haug et al. (2021) used individually tailored mobile-phone messages focusing on life skills; Debenham et al. (2022) evaluated an online neuroscience-based harm-reduction program; and Slade et al. (2023) combined an eHealth student program with parent-focused components. Follow-up periods also varied, ranging from 6 to 24–30 months, and the participants differed in baseline age and substance-use risk. Control conditions also differed across studies. Teesson et al. (2020), Debenham et al. (2022), and Slade et al. (2023) compared the intervention with usual or standard health education, whereas Haug et al. (2021) used an assessment-only control. Comparisons with routine health education may yield smaller incremental effects than comparisons with assessment-only or no-intervention controls because students in the former condition may receive substance-use or mental health-related content. Conversely, the intensity and content of routine health education were not standardized across trials, introducing additional between-study variation. These clinical and methodological differences may have contributed to variation in effect magnitude. Because only four studies were available, subgroup analyses by delivery format, follow-up duration, or control type would have resulted in subgroups containing only one or two studies and were therefore not performed. The corresponding forest plot is presented in Figure 2.

#### 3.3.2. Binge Drinking Outcomes

**Figure 3:**
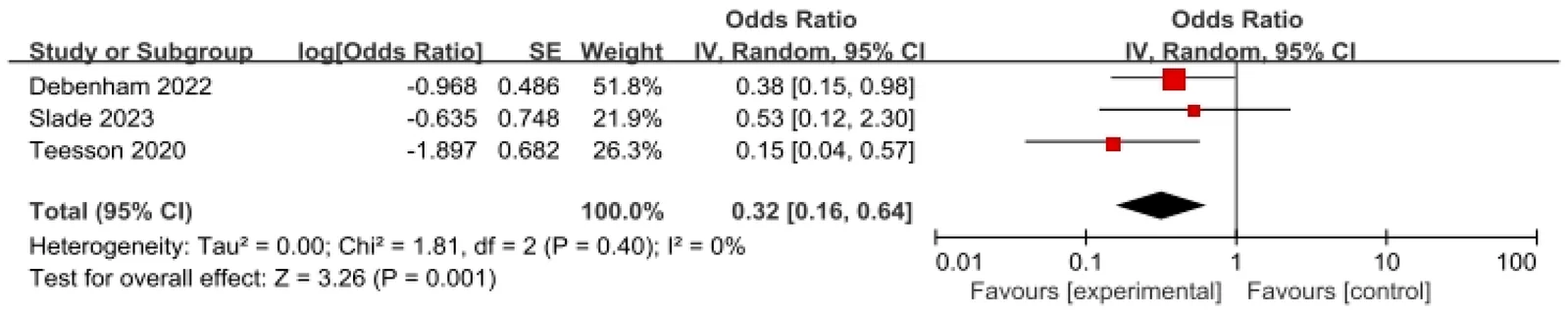
Forest plot of binge drinking outcomes (Teesson et al., 2020; Debenham et al., 2022; Slade et al., 2023).

The meta-analysis of binge drinking outcomes included three studies (Teesson et al., 2020; Debenham et al., 2022; Slade et al., 2023). Using a random-effects model, school-based integrated prevention programs significantly reduced the likelihood of binge drinking among adolescents compared with control groups (OR = 0.32, 95% CI [0.16, 0.64], *p* = 0.001). Individual effect sizes were strongest in Teesson et al. (2020) (OR = 0.15, 95% CI [0.04, 0.57]), followed by Debenham et al. (2022) (OR = 0.38, 95% CI [0.15, 0.98]) and Slade et al. (2023) (OR = 0.53, 95% CI [0.12, 2.30]). Although Slade et al. (2023) reported a non-significant effect estimate with a relatively wide confidence interval crossing the null value, the direction of effect remained consistent with the other included studies. No heterogeneity was observed across studies (I^2^ = 0%), indicating highly consistent intervention effects. Among all pooled outcomes examined in this study, binge drinking demonstrated the strongest and most consistent intervention effect. The forest plot for binge drinking outcomes is shown in Figure 3.

#### 3.3.3. Other Substance Use-Related Outcomes

Additional exploratory meta-analyses were conducted for other substance use-related outcomes, including cannabis use, tobacco-related outcomes, and intentions to use synthetic cannabis. This analysis included three studies (Champion et al., 2018; Haug et al., 2021; Debenham et al., 2022). The pooled effect size was not statistically significant (OR = 0.60, 95% CI [0.24, 1.47], *p* = 0.26).

At the individual study level, Champion et al. (2018) reported reduced intentions to use synthetic cannabis (OR = 0.28, 95% CI [0.10, 0.75]), while Debenham et al. (2022) demonstrated a reduction in high-risk cannabis use (OR = 0.48, 95% CI [0.25, 0.92]). In contrast, Haug et al. (2021) reported no statistically significant difference (OR = 1.24, 95% CI [0.89, 1.73]).

Considerable heterogeneity was observed across studies (I^2^ = 84%). Qualitative examination indicated that the pooled studies differed substantially in the substances and constructs assessed. Champion et al. (2018) evaluated intentions to use synthetic cannabis, Haug et al. (2021) assessed tobacco- and cannabis-related behaviors following a tailored mobile life-skills intervention, and Debenham et al. (2022) examined high-risk cannabis use following a neuroscience-based harm-reduction program. Thus, the analysis combined an intention-based outcome with behavioral outcomes involving different substances, baseline prevalence levels, and measurement instruments. The interventions also differed in modality, comprising a teacher-supervised online module, tailored mobile messaging, and an online classroom-based harm-reduction program, while follow-up periods ranged from 6 to 24 months. The control conditions also differed, with Champion et al. (2018) and Debenham et al. (2022) using health education as usual and Haug et al. (2021) using an assessment-only control. This difference may have influenced the magnitude of the observed effects because routine health education could itself contain relevant preventive content. These differences likely contributed to the marked variation in effect estimates. Because only three studies were included, subgroup analyses by intervention or control type would have consisted of single-study or two-study subgroups and were not considered methodologically informative. Therefore, this pooled estimate should be regarded as exploratory and interpreted with considerable caution. The forest plot for other substance-related outcomes is presented in Supplementary Figure S1.

### 3.4. Sensitivity and Exploratory Analyses

#### 3.4.1. Long-Term Follow-Up Analysis of Binge Drinking Outcomes

Sensitivity analyses including long-term follow-up data additionally incorporated Newton et al. (2022b) and Teesson et al. (2024). Although the pooled effect estimate numerically favored the intervention group (OR = 0.38, 95% CI [0.07, 1.93]), the wide confidence interval and substantial heterogeneity indicate considerable uncertainty regarding long-term intervention effectiveness. Substantial heterogeneity was observed across studies (I^2^ = 68%). This heterogeneity may be related to differences in follow-up duration across studies and increased attrition during long-term follow-up periods. In particular, longer follow-up periods may increase participant attrition and measurement uncertainty.

**Figure 4:**
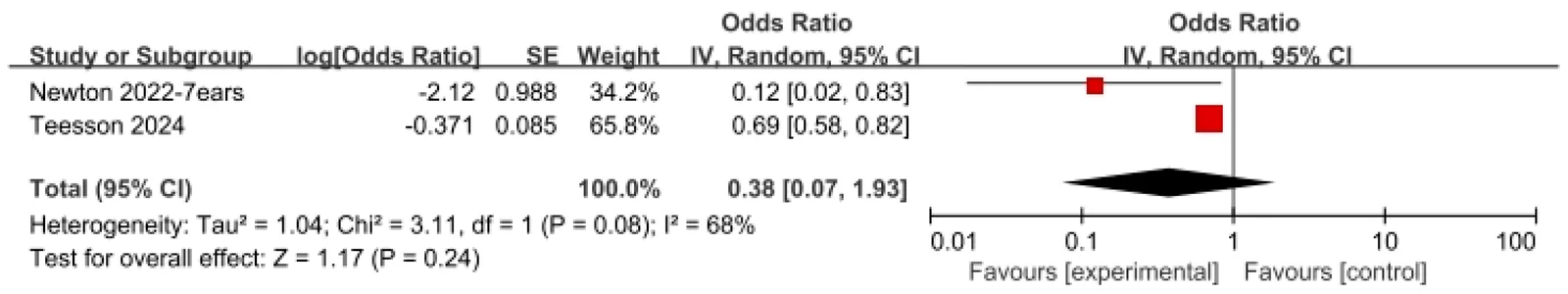
Forest plot of long-term follow-up sensitivity analysis (Newton et al., 2022b; Teesson et al., 2024).

Current evidence remains insufficient to determine whether intervention effects are sustained over the long term because of substantial heterogeneity and wide confidence intervals across studies. The forest plot for the long-term follow-up sensitivity analysis is presented in Figure 4.

#### 3.4.2. Tobacco-Related Outcomes

Additional analyses of tobacco- and nicotine-related outcomes were conducted primarily based on the study by Debenham et al. (2021), examining the outcomes of “any tobacco use” and “intentions to use tobacco.” The effect on actual tobacco use (“any tobacco use”) was significant (OR = 0.62, 95% CI [0.45, 0.85], Z = 2.95, *p* = 0.003), indicating a significant reduction in tobacco use among adolescents in the intervention group. Similarly, intentions to use tobacco were also significantly reduced (OR = 0.76, 95% CI [0.60, 0.96], Z = 2.29, *p* = 0.02).

The pooled overall effect size combining both outcomes was OR = 0.71 (95% CI [0.58, 0.85], Z = 3.57, *p* = 0.0004), with minimal heterogeneity observed across outcomes (I^2^ = 1%). The forest plot for tobacco-related outcomes is presented in Supplementary Figure S2. These findings suggest that school-based prevention programs may positively influence both actual tobacco use and intentions to use tobacco among adolescents. However, the findings should be interpreted cautiously because they were primarily based on a single study.

### 3.5. Risk of Bias

**Figure 5:**
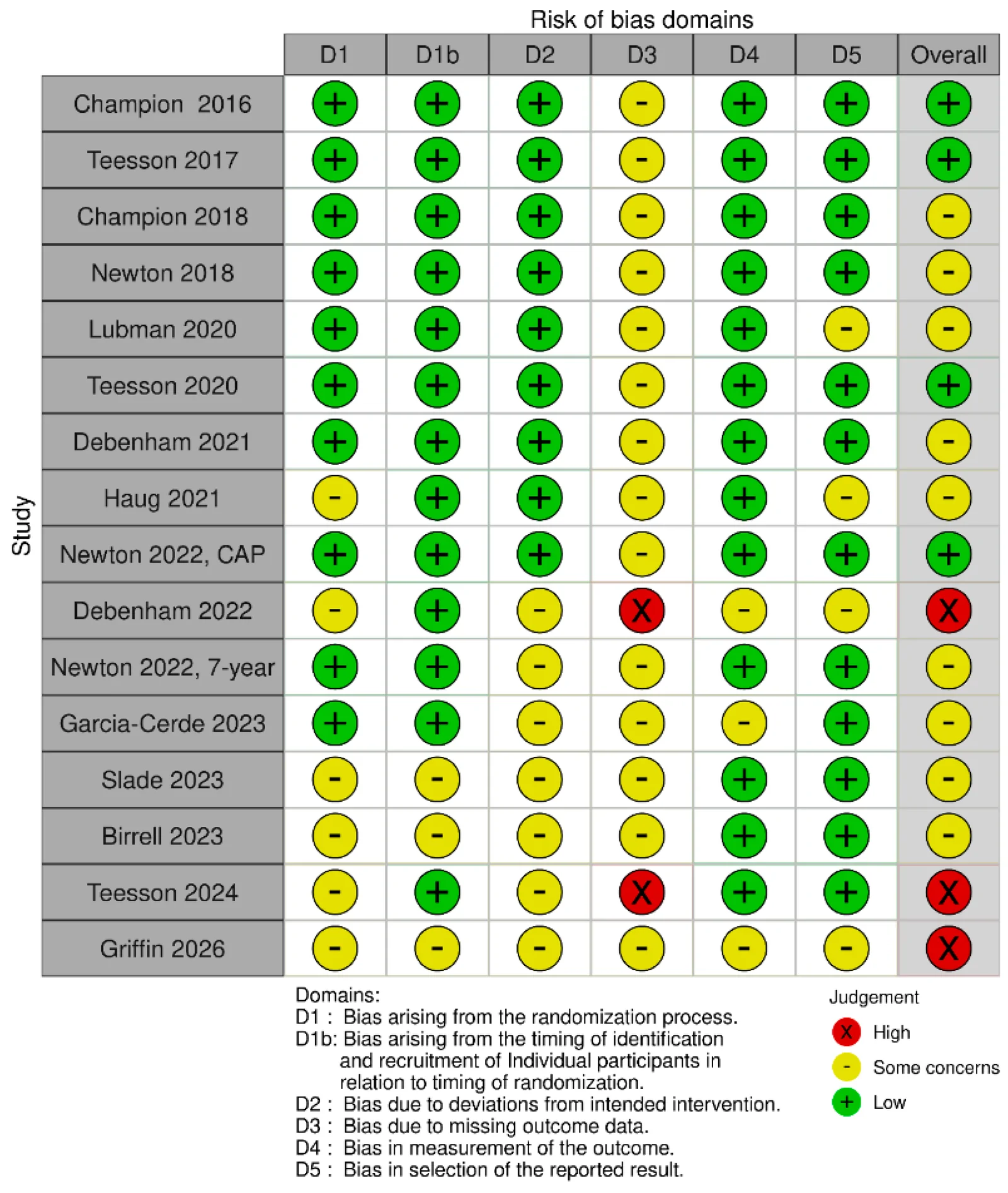
Risk of bias assessment using RoB 2 for cluster randomized trials (Champion et al., 2016, 2018; Teesson et al., 2017, 2020, 2024; Newton et al., 2018, 2022a, 2022b; Lubman et al., 2020; Debenham et al., 2021, 2022; Haug et al., 2021; Garcia-Cerde et al., 2023; Slade et al., 2023; Birrell et al., 2023; Griffin et al., 2026).

Risk of bias was assessed using the Cochrane RoB 2 tool for cluster randomized trials (RoB 2 for Cluster RCTs). The overall risk-of-bias assessment is presented in Figure 5.

Overall, most studies demonstrated low risk of bias in the domains of the randomization process (D1) and measurement of outcomes (D4). However, several studies, particularly those involving long-term follow-up assessments, were rated as having “some concerns” or higher in the domain of missing outcome data (D3).

In particular, Debenham et al. (2022), Teesson et al. (2024), and Griffin et al. (2026) were assessed as having high risk of bias due to missing outcome data because of high attrition rates and incomplete follow-up assessments. In addition, Newton et al. (2022a), Garcia-Cerde et al. (2023), Slade et al. (2023), Birrell et al. (2023), and Teesson et al. (2024) showed some concerns in the domain of deviations from intended interventions (D2).

Based on the overall risk-of-bias judgment, Champion et al. (2016), Teesson et al. (2017), Teesson et al. (2020), and Newton et al. (2022a) were classified as low risk of bias. In contrast, Debenham et al. (2022), Teesson et al. (2024), and Griffin et al. (2026) were classified as high risk of bias, whereas most of the remaining studies were categorized as having “some concerns.” Overall, the included school-based prevention intervention studies demonstrated a moderate risk-of-bias profile, with increased attrition and missing outcome data during long-term follow-up identified as major methodological limitations.

### 3.6. Publication Bias and Selective Reporting

Formal funnel-plot or regression-based assessments of publication bias were not conducted because the primary meta-analysis included only five independent trial comparisons, while the other pooled analyses included three or four studies. The RoB 2 assessment of selection of the reported results and qualitative examination of outcome-reporting completeness did not identify clear evidence that outcomes were omitted solely according to their direction or statistical significance. However, outcome definitions, assessment periods, and reporting completeness varied across studies, and unpublished findings could not be evaluated. Therefore, publication bias and selective reporting cannot be excluded.

## 4. Discussion

This systematic review and meta-analysis examined the effectiveness of school-based integrated mental health and substance use prevention programs for adolescents. Quantitative synthesis showed that integrated prevention programs were associated with significant reductions in alcohol-related outcomes and binge drinking among adolescents, whereas mental health and psychosocial outcomes were examined narratively and yielded less consistent evidence. In particular, binge drinking demonstrated the strongest and most consistent intervention effect across pooled analyses, with no observed heterogeneity between studies. These findings suggest that school-based integrated prevention programs incorporating mental health-related components may contribute to reducing adolescent substance use-related risk behaviors while strengthening psychosocial protective factors.

The current review extends previous school-based substance use prevention literature by emphasizing the potential role of integrated mental health-related components—such as resilience, emotional regulation, coping skills, self-regulation, help-seeking promotion, social competence, and psychosocial wellbeing—integrated into substance use prevention programs. Several interventions included in this review successfully incorporated these psychosocial and mental health-related strategies. For instance, programs combining universal substance prevention with emotional regulation or cognitive–behavioral coping components, such as the Climate and Preventure (CAP) studies and Climate Schools Combined (CSC), have laid a strong foundation for addressing overlapping risk factors. These integrated approaches may strengthen adolescents’ ability to manage emotional distress and peer-related pressures without engaging in substance use behaviors.

Among all pooled outcomes, binge drinking demonstrated the strongest and most consistent intervention effect. This finding may reflect the suitability of integrated psychosocial interventions for modifying social and behavioral mechanisms associated with high-risk alcohol consumption patterns. Universal online and hybrid interventions targeting self-regulation, drug literacy, and emotional coping appear particularly effective. For example, the neuroscience-based harm reduction framework evaluated in the Illicit Project (Debenham et al., 2022) significantly targeted high-risk consumption, while the Climate Schools Plus (CSP) model (Slade et al., 2023) demonstrated the utility of structured eHealth prevention in reducing risky drinking patterns.

The findings of this review also suggest that integrated prevention programs may positively influence broader psychosocial outcomes beyond substance use behaviors alone, with several studies reporting narrative or secondary improvements in emotional regulation, resilience, self-control, social competence, and help-seeking behaviors. Importantly, personality-targeted preventive interventions have demonstrated significant broader developmental benefits; for instance, Lawler et al. (2024) underscored the long-term effectiveness of these programs in mitigating behavioral challenges, showing sustained reductions in outcomes such as aggression from adolescence into early adulthood. Although secondary mental health-related outcomes such as depression and anxiety were frequently reported, quantitative synthesis for these outcomes was not conducted due to substantial heterogeneity in measurement tools, assessment time points, and intervention characteristics. Nevertheless, the narrative findings suggest that integrated prevention programs may contribute to strengthening psychosocial wellbeing and emotional functioning among adolescents.

Peer-based approaches may provide an important complementary strategy for school-based integrated prevention because peer norms, modeling, and social influence can shape adolescents’ attitudes toward substance use and help-seeking. Although most programs included in this review were delivered by teachers, psychologists, or digital platforms rather than through formal peer education, some incorporated peer-related mechanisms. For example, Mind Your Mate used a peer-support mobile application to address mental health, substance use, and help-seeking, while #Tamojunto2.0 targeted alcohol-related beliefs and social influences within the school context. Compared with professionally or digitally delivered programs, peer-based approaches may enhance relatability, credibility, and student engagement; however, they should not be viewed as replacements for structured evidence-based interventions. Instead, trained peer facilitators or peer-support components could complement teacher-led and digital programs by reinforcing healthy norms, facilitating early help-seeking, and extending prevention messages through adolescents’ social networks. Future studies should directly compare peer-led, professionally delivered, and combined delivery models while examining the training, supervision, fidelity, and safety procedures required for effective peer involvement.

Several included trials reported intervention-related changes from baseline relative to control conditions in substance-related knowledge, beliefs, intentions, and study-specific psychosocial outcomes, even when reductions in actual substance use were not consistently observed. The term “psychosocial outcomes” refers to separately measured constructs—including perceived stress, emotional regulation, wellbeing, social competence, and confidence in help-seeking—rather than a composite protective-factor score calculated in this review. Evidence regarding the persistence of these effects was mixed. Improvements in substance-related knowledge or intentions were maintained for up to 24 months in the Climate Schools: Ecstasy and Emerging Drugs trial (Champion et al., 2018), and knowledge gains were observed through 30 months in the Climate Schools Combined trial (Teesson et al., 2020). In contrast, improvements in confidence to support peer help-seeking in MAKINGtheLINK were evident primarily at the 6-week and 6-month assessments and were not consistently maintained at 12 months (Lubman et al., 2020). Furthermore, the long-term follow-up of Climate Schools Combined did not demonstrate sustained mental health effects beyond the earlier follow-up periods (Teesson et al., 2024). Therefore, cognitive and psychosocial changes may precede or support behavioral change, but the current evidence does not establish that these outcomes are consistently sustained over the long term.

Furthermore, this review highlighted the growing use and viability of digital and eHealth-based interventions, with many included studies evaluating online, app-based, mobile phone–delivered, or web-based prevention programs. Digital interventions—such as Climate Schools models (Champion et al., 2016, 2018), SmartCoach (Haug et al., 2021), and the peer-support mobile app Mind Your Mate (Birrell et al., 2023)—offer several advantages, including scalability, accessibility, implementation consistency, and cost-effectiveness. Technology-based interventions can provide interactive and individualized content while reducing barriers associated with stigma and limited access to mental health resources. In the current review, digital interventions frequently improved alcohol-related knowledge, drug literacy, harm-reduction skills, and specific alcohol-related outcomes. Furthermore, digital interventions may help overcome common barriers associated with traditional face-to-face prevention programs, including limited teacher training, inconsistent implementation fidelity, and restricted access to specialized professionals. Given the global burden of adolescent mental health problems and substance use, scalable digital prevention strategies may become increasingly important in school and public health settings.

The current review also emphasized the importance of implementation quality, school engagement, and contextual adaptation. Several studies suggested that interventions with stronger implementation support, standardized delivery platforms, and higher fidelity demonstrated more favorable outcomes. Conversely, limited teacher time, heavy workloads, competing academic priorities, and insufficient institutional support frequently emerged as barriers to sustainable implementation. These findings suggest that effective dissemination of school-based prevention programs requires not only evidence-based intervention content but also sustainable implementation infrastructure and organizational support. Cultural adaptation and contextual relevance also appeared to influence intervention acceptability and feasibility. Although most studies were conducted in high-income countries, interventions implemented across diverse international settings—such as #Tamojunto2.0 in Brazil (Garcia-Cerde et al., 2023), SmartCoach in Switzerland (Haug et al., 2021), and the LST-HS Hybrid program in the USA (Griffin et al., 2026)—demonstrated that tailored prevention programs can optimize student engagement and implementation feasibility across distinct educational landscapes. Therefore, future dissemination efforts should carefully consider cultural, linguistic, and educational adaptation processes.

Although 16 reports were included, these represented 10 independent parent trials because several articles were follow-up or secondary analyses of the same participant cohorts. In particular, Champion et al. (2016) and Champion et al. (2018) reported different follow-up periods from the same trial, while multiple publications from the CAP and Climate Schools Combined trials examined distinct outcomes or extended follow-up periods. Retaining these reports enabled a more complete assessment of outcome domains and intervention sustainability; however, the number of publications should not be interpreted as the number of independent evidence sources. To prevent double-counting, only one effect estimate from each parent trial was included for a given outcome and assessment period within each meta-analysis. Consequently, removing overlapping earlier reports would reduce descriptive detail but would not materially alter the pooled conclusions because overlapping participant data were not counted more than once within the same quantitative synthesis. Nevertheless, the concentration of evidence within a relatively small number of parent trials limits the breadth and independence of the evidence base.

Despite these promising findings, several limitations should be considered. First, substantial heterogeneity was observed in the pooled analyses of alcohol-related outcomes (I^2^ = 60%) and other substance-related outcomes (I^2^ = 84%). Qualitative examination suggested that this heterogeneity reflected differences in the substances and outcome constructs assessed, intervention components and delivery formats, control conditions, participant age and baseline risk, measurement instruments, and follow-up durations. In particular, the included studies used control conditions ranging from no intervention or assessment only to routine or standard health education. Effect estimates derived from comparisons with no-intervention or assessment-only controls may appear larger than those derived from comparisons with routine health education, which may itself include substance-use prevention or mental health-related content. However, the content and intensity of routine health education also varied and were not consistently reported. Accordingly, the pooled estimates represent intervention effects across heterogeneous comparator conditions rather than effects relative to a single standardized control. The analysis of other substance-related outcomes was particularly heterogeneous because it combined an intention-based outcome with behavioral outcomes involving cannabis, tobacco, and other substances. In addition, the concept of integrated prevention varied across studies because the interventions incorporated different psychosocial and mental health-related components. Because only three or four studies were available for these analyses, reliable subgroup analyses by control type or other study characteristics and meta-regression could not be performed. Consequently, the pooled estimates—particularly for other substance-related outcomes—should be interpreted as exploratory rather than as evidence of a uniform intervention effect. Second, publication bias could not be formally assessed because each meta-analysis included fewer than 10 independent studies. Although the RoB 2 assessment and qualitative examination of reporting completeness did not identify clear evidence of selective outcome reporting, the small evidence base and inability to evaluate unpublished findings mean that publication and small-study biases cannot be excluded. Therefore, the pooled effects should be interpreted cautiously. Third, although significant pooled effects were observed for alcohol-related outcomes and binge drinking, overall effect sizes were generally modest, which may be partially explained by low baseline prevalence of substance use, implementation variability, or insufficient statistical power in certain sub-analyses. Fourth, most studies relied on self-reported measures of substance use and psychosocial functioning, which may be vulnerable to recall bias and social desirability bias. Finally, the geographical distribution of the evidence was markedly uneven, with a substantial proportion of the included studies conducted in Australia. Although studies from Switzerland, Brazil, and the United States were also represented, this concentration may introduce geographical bias and limit the generalizability of the findings to educational and health-care systems with different cultural contexts, school structures, prevention infrastructures, and patterns of adolescent substance use. Therefore, the findings should be interpreted cautiously when applied to regions that were underrepresented or not represented in the current evidence base.

Moreover, reporting of participant diversity and school or community context—including race or ethnicity, socioeconomic characteristics, and rural–urban location—was inconsistent across the included studies. This limited our ability to determine whether intervention effects differed across population groups and educational settings and further constrains the generalizability of the findings.

To fully clarify the sustainability of these interventions, rigorous long-term follow-up evaluations are critical. The sensitivity analyses incorporating multi-year follow-ups—such as the 7-year outcomes of the CAP study (Newton et al., 2022b) and the 72-month follow-up of Climate Schools Combined (Teesson et al., 2024)—revealed wide confidence intervals and increased uncertainty over time, partly reflecting participant attrition and missing outcome data at later assessments. The apparent attenuation of preventive effects over time may reflect both developmental processes and intervention-design limitations, although the available evidence does not permit their relative contributions to be determined. As adolescents progress toward early adulthood, ongoing neurocognitive maturation, increasing autonomy, changing peer networks and social norms, and greater exposure to substance-use opportunities may weaken skills or protective effects established during early adolescence. At the same time, many included interventions were delivered over a relatively brief period, and systematic booster sessions or developmentally adapted follow-up components were uncommon. Consequently, intervention content delivered at one developmental stage may become less salient as adolescents encounter new social contexts and risks. These findings suggest that sustained prevention may require periodic booster sessions, age-appropriate adaptation of content, and reinforcement through peer, family, and school systems during key developmental transitions. Future trials should assess potential developmental mediators and compare programs with and without booster components to distinguish developmental attenuation from limitations in intervention duration and reinforcement.

Additional research is needed to evaluate culturally adaptable, scalable, and sustainable integrated prevention approaches across diverse sociocultural and educational settings, ensuring that improvements in psychosocial protective factors, behavior regulation, and reductions in substance use-related risk behaviors are effectively maintained from mid-adolescence into early adulthood.

## 5. Conclusions

The findings of this systematic review and meta-analysis suggest that school-based integrated prevention programs incorporating mental health-related components may provide a promising approach for reducing adolescent substance use-related risk behaviors while strengthening psychosocial protective factors and emotional wellbeing. Interventions targeting emotional regulation, coping skills, resilience, social competence, self-regulation, and help-seeking behaviors demonstrated beneficial effects across several substance use-related outcomes, particularly alcohol-related behaviors and binge drinking. Digital and eHealth-based interventions also emerged as potentially scalable and accessible approaches for adolescent substance use prevention within school settings. These approaches may improve accessibility, implementation consistency, and student engagement while reducing barriers associated with traditional face-to-face delivery models.

However, the overall evidence remains heterogeneous, and intervention effects were generally modest. Program effectiveness appeared to depend heavily on implementation quality, intervention fidelity, contextual adaptation, and sustained engagement from schools, facilitators, students, and families. Future research should prioritize multicenter studies conducted across diverse geographical, cultural, and educational settings, using standardized outcome measures and longer follow-up periods to strengthen the external validity and comparability of the evidence. Such studies should also examine implementation fidelity and identify the psychosocial and mental health-related components that are most effective in preventing adolescent substance use-related risk behaviors.

## Figures and Tables

**Table 1:** Characteristics of the reports included in the systematic review and meta-analysis.

Authors (Country)	N (Female %)	Mean Age	Intervention Name	Intervention Type	Integrated Components	Delivery Mode	Follow-Up
Champion et al. (2016) (Australia)	EG = 636 CG = 490 (43%)	14.9	Climate Schools: Ecstasy and Emerging Drugs	Universal	Substance use (Ecstasy/NPS knowledge, harm minimisation)	Online	Post-test, 6 mo, 12 mo
Teesson et al. (2017) (Australia)	2190 (57.6%)	13.3	CAP Study (Climate Schools + Preventure)	Universal + Selective (Combined)	Alcohol use + emotional regulation	Online + face-to-face	6 mo, 12 mo, 24 mo
Champion et al. (2018) (Australia)	1126 (43%)	14.9	Climate Schools: Ecstasy and Emerging Drugs (24-month FU)	Universal	Substance use (Ecstasy/NPS)	Online	Post-test, 6 mo, 12 mo, 24 mo
Newton et al. (2018) (Australia)	2190 (50%)	13.3	CAP Study: Cannabis outcomes	Universal + Selective (Combined)	Cannabis knowledge and use	Online	6 mo, 12 mo, 24 mo, 36 mo
Lubman et al. (2020) (Australia)	2447 (50%)	14.9	MAKINGtheLINK	Universal	Mental health literacy + help-seeking	Face-to-face	6 weeks, 6 mo, 12 mo
Teesson et al. (2020) (Australia)	6386 (54.8%)	13.5	Climate Schools Combined (CSC)	Universal (Combined SU + MH)	Alcohol/cannabis + depression/anxiety	Online	6 mo, 12 mo, 24 mo, 30 mo
Newton et al. (2022a) (Australia)	6386 (54.8%)	13.5	Climate Schools: Alcohol and Cannabis	Universal	Alcohol + cannabis prevention	Online	6 mo, 12 mo
Debenham et al. (2021) (Australia)	1005 (44%)	13.4	Preventure Tobacco outcomes	Selective	Tobacco prevention + emotional regulation	Face-to-face	6 mo, 12 mo, 24 mo, 36 mo
Haug et al. (2021) (Switzerland)	1473 (55.2%)	15.4	SmartCoach	Universal	Alcohol/tobacco/cannabis + life skills	Mobile/web-based	6 mo, 18 mo
Debenham et al. (2022) (Australia)	950 (60%)	15.9	The Illicit Project	Universal	Alcohol/cannabis/MDMA/nicotine + drug literacy	Online	6 mo
Newton et al. (2022b) (Australia)	2190 (42.6%)	13.3	CAP Study: 7-year follow-up	Universal + Selective (Combined)	Alcohol prevention + coping/MH	Online + face-to-face	5.5 yr, 7 yr
Garcia-Cerde et al. (2023) (Brazil)	5208 (49.4%)	13.2	#Tamojunto2.0	Universal	Alcohol, binge drinking, tobacco, marijuana + life skills	Face-to-face	9 mo
Slade et al. (2023) (Australia)	562 (59.1%)	13.5	Climate Schools Plus (CSP)	Universal	Alcohol prevention + parent engagement	Online	12 mo, 24 mo
Birrell et al. (2023) (Australia)	166 (43.4%)	15.3	Mind Your Mate	Universal	Mental health + substance use + help-seeking	Blended	6 mo, 12 mo
Teesson et al. (2024) (Australia)	6386 (54.8%)	13.5	Climate Schools Combined (CSC)—72-month FU	Universal (Combined SU + MH)	Alcohol/cannabis + depression/anxiety	Online	60 mo, 72 mo
Griffin et al. (2026) (USA)	1235 (51%)	15.2	LST-HS Hybrid	Universal	Prescription drug misuse + life skills	Blended/Hybrid	Post-test

Note. EG = experimental group; CG = control group; FU = follow-up; SU = substance use; MH = mental health; NPS = new psychoactive substances.

## Data Availability

The data presented in this study are available within the article and Supplementary Materials.

## Supplementary Materials

The following supporting information can be downloaded at: https://www.mdpi.com/article/10.3390/bs16091498/s1, Supplementary Figure S1. Forest plot of other substance-related outcomes; Supplementary Figure S2. Forest plot of tobacco-related outcomes; Supplementary Table S1. Searching Strategy (PubMed); Supplementary Table S2. PRISMA 2020 Checklist (Page et al., 2021); Supplementary Table S3. Additional Intervention, Comparator, and Contextual Characteristics of the Included Studies.
