# Supplementary material for: Effectiveness of School-Based Integrated Mental Health and Substance Use Prevention Programs for Adolescents: A Systematic Review and Meta-Analysis
Source: Behav Sci (Basel). 2026 Aug 26;16(9):1498. doi: 10.3390/bs16091498 (PMC13603272; doi:10.3390/bs16091498)
Supplement: Supplementary file 1 [file behavsci-16-01498-s001.zip › behavsci-4401555-supplementary.pdf]

Study or Subgroup	log[Odds Ratio]	SE	Weight	Odds Ratio IV, Random, 95% CI
Champion 2018	-1.27	0.503	27.5%	0.28 [0.10, 0.75]
Debenham 2022	-0.734	0.333	33.7%	0.48 [0.25, 0.92]
Haug 2021	0.215	0.17	38.8%	1.24 [0.89, 1.73]
**Total (95% CI)**			**100.0%**	**0.60 [0.24, 1.47]**

Heterogeneity: $Tau^2 = 0.51$; $Chi^2 = 12.46$, df = 2 (P = 0.002); $I^2 = 84\%$
Test for overall effect: Z = 1.12 (P = 0.26)

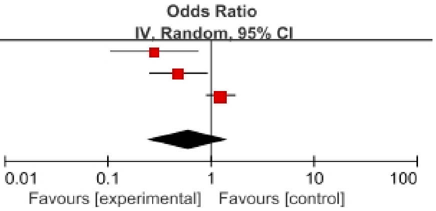

Supplementary Figure S1. Forest plot of other substance-related outcomes

Study or Subgroup	log[Odds Ratio]	SE	Weight	Odds Ratio IV, Random, 95% CI
**2.3.1 Any tobacco use**				
Debenham 2021	-0.478	0.162	35.6%	0.62 [0.45, 0.85]
**Subtotal (95% CI)**			**35.6%**	**0.62 [0.45, 0.85]**
Heterogeneity: Not applicable				
Test for overall effect: Z = 2.95 (P = 0.003)				
**2.3.2 Intentions to use tobacco**				
Debenham 2021	-0.275	0.12	64.4%	0.76 [0.60, 0.96]
**Subtotal (95% CI)**			**64.4%**	**0.76 [0.60, 0.96]**
Heterogeneity: Not applicable				
Test for overall effect: Z = 2.29 (P = 0.02)				
**Total (95% CI)**			**100.0%**	**0.71 [0.58, 0.85]**

Heterogeneity: $Tau^2 = 0.00$; $Chi^2 = 1.01$, df = 1 (P = 0.31); $I^2 = 1\%$
Test for overall effect: Z = 3.57 (P = 0.0004)
Test for subgroup differences: $Chi^2 = 1.01$, df = 1 (P = 0.31), $I^2 = 1.4\%$

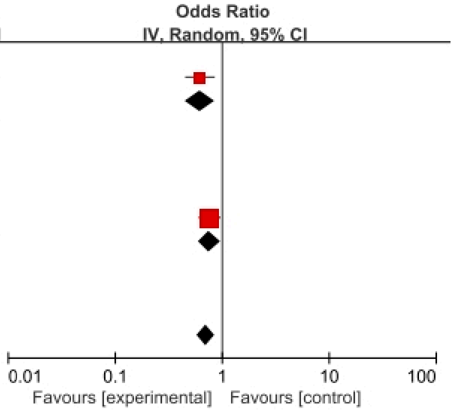

Supplementary Figure S2. Forest plot of tobacco-related outcomes

Supplementary Table S1. Searching Strategy (PubMed)

No.	Searching term
1	Adolescent [Mesh]
2	Adolescent* OR Teen* OR Youth* OR "High school student*" OR "Middle school student*"
3	1 OR 2
4	Schools [Mesh]
5	"School-based" OR "School program*" OR "School intervention*" OR "Educational setting" OR Classroom*
6	4 OR 5
7	Substance-Related Disorders [Mesh]
8	"Substance use" OR "Drug use" OR "Substance abuse" OR Alcohol* OR Smoking OR Tobacco OR Vaping OR Cannabis OR Marijuana
9	7 OR 8
10	Mental Health [Mesh]
11	"Mental health" OR Depression OR Anxiety OR Stress OR Resilience OR "Psychological well-being" OR "Emotional well-being" OR "School adjustment" OR "Self-esteem"
12	10 OR 11
13	Prevention and Control[Mesh] OR Health Promotion [Mesh] OR Primary Prevention [Mesh]
14	Prevention OR Intervention* OR Program* OR "Preventive intervention*" OR "Health education"
15	13 OR 14
16	3 AND 6 AND 9 AND 12 AND 15

Supplementary Table S2. PRISMA 2020 Checklist

Section and Topic	Item #	Checklist item	Location where item is reported
**TITLE**			
Title	1	Identify the report as a systematic review.	page1
**ABSTRACT**			
Abstract	2	See the PRISMA 2020 for Abstracts checklist.	page 2-4
**INTRODUCTION**			
Rationale	3	Describe the rationale for the review in the context of existing knowledge.	page 3-4
Objectives	4	Provide an explicit statement of the objective(s) or question(s) the review addresses.	page 4
**METHODS**			
Eligibility criteria	5	Specify the inclusion and exclusion criteria for the review and how studies were grouped for the syntheses.	page 6-7
Information sources	6	Specify all databases, registers, websites, organisations, reference lists and other sources searched or consulted to identify studies. Specify the date when each source was last searched or consulted.	page 5
Search strategy	7	Present the full search strategies for all databases, registers and websites, including any filters and limits used.	page 5
Selection process	8	Specify the methods used to decide whether a study met the inclusion criteria of the review, including how many reviewers screened each record and each report retrieved, whether they worked independently, and if applicable, details of automation tools used in the process.	page 6
Data collection process	9	Specify the methods used to collect data from reports, including how many reviewers collected data from each report, whether they worked independently, any processes for obtaining or confirming data from study investigators, and if applicable, details of automation tools used in the process.	page 7
Data items	10a	List and define all outcomes for which data were sought. Specify whether all results that were compatible with each outcome domain in each study were sought (e.g. for all measures, time points, analyses), and if not, the methods used to decide which results to collect.	page 7
	10b	List and define all other variables for which data were sought (e.g. participant and intervention characteristics, funding sources). Describe any assumptions made about any missing or unclear information.	page 7
Study risk of bias assessment	11	Specify the methods used to assess risk of bias in the included studies, including details of the tool(s) used, how many reviewers assessed each study and whether they worked independently, and if applicable, details of automation tools used in the process.	page 8
Effect	12	Specify for each outcome the effect measure(s) (e.g. risk ratio, mean difference) used in the synthesis or presentation of	page 9

Section and Topic	Item #	Checklist item	Location where item is reported
measures		results.	
Synthesis methods	13a	Describe the processes used to decide which studies were eligible for each synthesis (e.g. tabulating the study intervention characteristics and comparing against the planned groups for each synthesis (item #5)).	page 6
	13b	Describe any methods required to prepare the data for presentation or synthesis, such as handling of missing summary statistics, or data conversions.	page 9
	13c	Describe any methods used to tabulate or visually display results of individual studies and syntheses.	page 9-9
	13d	Describe any methods used to synthesize results and provide a rationale for the choice(s). If meta-analysis was performed, describe the model(s), method(s) to identify the presence and extent of statistical heterogeneity, and software package(s) used.	page 9
	13e	Describe any methods used to explore possible causes of heterogeneity among study results (e.g. subgroup analysis, meta-regression).	page 9
	13f	Describe any sensitivity analyses conducted to assess robustness of the synthesized results.	page 9
Reporting bias assessment	14	Describe any methods used to assess risk of bias due to missing results in a synthesis (arising from reporting biases).	page 9
Certainty assessment	15	Describe any methods used to assess certainty (or confidence) in the body of evidence for an outcome.	Not assessed
**RESULTS**			
Study selection	16a	Describe the results of the search and selection process, from the number of records identified in the search to the number of studies included in the review, ideally using a flow diagram.	page 9-10
	16b	Cite studies that might appear to meet the inclusion criteria, but which were excluded, and explain why they were excluded.	page 9-10
Study characteristics	17	Cite each included study and present its characteristics.	page 10
Risk of bias in studies	18	Present assessments of risk of bias for each included study.	page 11
Results of individual studies	19	For all outcomes, present, for each study: (a) summary statistics for each group (where appropriate) and (b) an effect estimate and its precision (e.g. confidence/credible interval), ideally using structured tables or plots.	page 10-11
Results of syntheses	20a	For each synthesis, briefly summarise the characteristics and risk of bias among contributing studies.	page 10-11
	20b	Present results of all statistical syntheses conducted. If meta-analysis was done, present for each the summary estimate and its precision (e.g. confidence/credible interval) and measures of statistical heterogeneity. If comparing groups, describe the direction of the effect.	page 11-12

Section and Topic	Item #	Checklist item	Location where item is reported
	20c	Present results of all investigations of possible causes of heterogeneity among study results.	page 11-12
	20d	Present results of all sensitivity analyses conducted to assess the robustness of the synthesized results.	page 12-13
Reporting biases	21	Present assessments of risk of bias due to missing results (arising from reporting biases) for each synthesis assessed.	page 13-14
Certainty of evidence	22	Present assessments of certainty (or confidence) in the body of evidence for each outcome assessed.	Not assessed
**DISCUSSION**			
Discussion	23a	Provide a general interpretation of the results in the context of other evidence.	page 14
	23b	Discuss any limitations of the evidence included in the review.	page 15-16
	23c	Discuss any limitations of the review processes used.	page 17-18
	23d	Discuss implications of the results for practice, policy, and future research.	page 19-20
**OTHER INFORMATION**			
Registration and protocol	24a	Provide registration information for the review, including register name and registration number, or state that the review was not registered.	page 5
	24b	Indicate where the review protocol can be accessed, or state that a protocol was not prepared.	page 5
	24c	Describe and explain any amendments to information provided at registration or in the protocol.	No amendments were made to the registered protocol.
Support	25	Describe sources of financial or non-financial support for the review, and the role of the funders or sponsors in the review.	This study did not receive any specific financial support from public, commercial, or non-profit funding agencies. No funders or sponsors had any role in the

Section and Topic	Item #	Checklist item	Location where item is reported
			design, conduct, analysis, or reporting of the review.
Competing interests	26	Declare any competing interests of review authors.	The authors declare no competing interests.
Availability of data, code and other materials	27	Report which of the following are publicly available and where they can be found: template data collection forms; data extracted from included studies; data used for all analyses; analytic code; any other materials used in the review.	All data analyzed during this study are included in this published article and its supplementary materials. Additional data are available from the corresponding author upon reasonable

From: Page MJ, McKenzie JE, Bossuyt PM, Boutron I, Hoffmann TC, Mulrow CD, et al. The PRISMA 2020 statement: an updated guideline for reporting systematic reviews. BMJ 2021;372:n71. doi: 10.1136/bmj.n71. This work is licensed under CC BY 4.0. To view a copy of this license, visit https://creativecommons.org/licenses/by/4.0/

Supplementary Table S3. Additional Intervention, Comparator, and Contextual Characteristics of the Included Studies

Authors (Country)	Duration/Sessions	Delivered By	Control Condition	Participating Schools /School Type	Geographical /Community Setting	Cultural/Racial /Ethnic Characteristics	Socioeconomic Characteristics	Parent Trial /Notes
Champion et al. (2016) (Australia)	4 lessons × 40 min over 4 weeks	Teacher-supervised	Health education as usual	11 secondary schools; all independent	Sydney, New South Wales	Country of birth assessed; race/ethnicity not reported	Not reported	Original Climate Schools: Ecstasy and Emerging Drugs trial
Teesson et al. (2017) (Australia)	12 lessons × 40 min + 2 × 90 min sessions	Teacher + psychologist	Health education as usual	26 secondary schools; 17 private and 9 public	New South Wales and Victoria; rural-urban status not reported	Country of birth assessed; predominantly Australian-born; race/ethnicity not reported	Not reported for the overall sample	CAP parent trial
Champion et al. (2018) (Australia)	4 lessons × 40 min over 4 weeks	Teacher-supervised	Health education as usual	11 secondary schools; all independent	Sydney, New South Wales	Country of birth assessed; race/ethnicity not reported	Socioeconomic status considered in adjusted analyses, but its distribution was not reported	24-month follow-up of Champion et al. (2016)
Newton et al. (2018) (Australia)	12 lessons × 40 min + 2 × 90 min sessions	Teacher + psychologist	Health education as usual	19 schools in the Control, Climate, and CAP groups; male-only, female-only, and coeducational schools	New South Wales and Victoria; rural-urban status not reported	Born in Australia 86.9%; other English-speaking countries 6.8%; non-English-speaking countries 6.2%; race/ethnicity not reported	Not reported	Cannabis-outcome analysis from the CAP parent trial; standalone Preventure arm excluded
Lubman et al. (2020) (Australia)	5 activities over 2 class periods + 1 booster session	Teacher-facilitated	Waitlist control	21 secondary schools at baseline;	Within 50 km of Melbourne's central	English was the primary home language for 84%;	Mean ICSEA scores were 1034 in the	MAKINGtheLINK trial

				Government, Catholic, and Independent schools	business district, Victoria	Vietnamese 3% and Arabic 2%; race/ethnicity not reported	control group and 1024 in the intervention group	
Teesson et al. (2020) (Australia)	12 substance-use + 6 mental-health lessons	Teacher	Active control	71 secondary schools; public, independent, and Catholic schools	New South Wales, Queensland, and Western Australia; metropolitan, coastal, and regional areas	Country of birth assessed; detailed race/ethnicity not reported	Participants were generally from schools located in areas of above-average socioeconomic status	CSC parent trial
Newton et al. (2022a) (Australia)	12 lessons × 40 min	Teacher	Health education as usual	71 secondary schools; public 52.6%, private 22.6%, and Catholic 24.8%	New South Wales, Queensland, and Western Australia; metropolitan, coastal, and regional areas	Country of birth assessed; race/ethnicity not reported	Detailed socioeconomic distribution not reported	Secondary analysis of the CSC parent trial
Debenham et al. (2021) (Australia)	2 × 90 min sessions	Psychologist + co-facilitator	Health education as usual	14 schools; 7 Preventure and 7 control schools; predominantly private-school participants	New South Wales and Victoria; rural-urban status not reported	Born in Australia 88.1%; race/ethnicity not reported	Participants were from schools with above-average socioeconomic advantage according to ICSEA	Tobacco-outcome analysis from the CAP parent trial
Haug et al. (2021) (Switzerland)	Tailored messages delivered over 22 weeks	Automated system	Assessment-only control	Number of schools not reported; 89 secondary and upper-secondary school classes	German-speaking Switzerland; rural-urban status not reported	No immigration background 47.4%; one parent born outside Switzerland 23.2%; both parents born outside Switzerland 29.5%	Not reported	SmartCoach trial; convenience sample of participating classes

Debenham et al. (2022) (Australia)	3 classroom sessions	Teacher-supervised	Health education as usual	8 secondary schools; 6 state and 2 independent schools	Locations across New South Wales; rural-urban status not reported	Born in Australia 94%; race/ethnicity not reported	Academic grades reported; direct socioeconomic indicators not reported	The Illicit Project trial
Newton et al. (2022b) (Australia)	12 lessons × 40 min + 2 × 90 min sessions	Teacher + psychologist	Health education as usual	26 schools in the original trial; 17 private and 9 public schools	New South Wales and Victoria; rural-urban status not reported	Country of birth reported; race/ethnicity not reported	Not reported	Seven-year follow-up of the CAP parent trial
Garcia-Cerde et al. (2023) (Brazil)	12 weekly lessons × 50 min	Trained teachers	No intervention	73 public middle schools; 36 intervention and 37 control schools	São Paulo, Fortaleza, and Eusébio; explicit rural-urban classification not reported	Race/ethnicity not reported	ABEP category A 3.4%, B 24.6%, C 53.9%, and D/E 16.9%	#Tamojunto2.0 trial
Slade et al. (2023) (Australia)	12 student lessons + parent webinars/modules	Teacher + facilitator	Health education as usual	12 secondary schools; 6 Catholic and 6 independent schools	Eleven schools in New South Wales and one in Queensland; rural-urban status not reported	Born in Australia 90.6%; other English-speaking countries 2.7%; non-English-speaking countries 6.7%; race/ethnicity not reported	Not reported	Climate Schools Plus trial
Birrell et al. (2023) (Australia)	1 classroom lesson + self-directed app use	Teacher-facilitated	Health education as usual	12 secondary schools; independent and public schools	Greater Sydney, New South Wales	Born in Australia 80.1%; born in another country 19.9%; race/ethnicity not reported	Not reported	Mind Your Mate trial
Teesson et al. (2024) (Australia)	18 lessons in the original intervention	Teacher	Health education as usual	71 secondary schools in the original	New South Wales, Queensland, and Western Australia;	Detailed ethnicity data were not collected because	Participants were generally recruited from	60- and 72-month follow-up of

				trial; public, independent, and Catholic schools	metropolitan, coastal, and regional areas	ethical approval did not permit collection	schools located in areas of above-average socioeconomic status in the original trial	the CSC parent trial
Griffin et al. (2026) (USA)	8 e-learning modules + 8 classroom sessions	E-learning platform + teacher	Standard health education	19 high schools; 10 intervention and 9 control schools	Northeast 43.8%, Midwest 31.2%, South 12.5%, and West 12.5%; rural-urban status not reported	White 59%, Black 10%, Asian 3%, mixed/other 8%, and Latino/Hispanic 25%	Approximately 30% received free or reduced-price lunch	LST-HS Hybrid trial

Note. ABEP = Brazilian Association of Research Companies; CAP = Climate and Preventure; CSC = Climate Schools Combined; ICSEA = Index of Community Socio-Educational Advantage. "Not reported" indicates that the relevant information was not provided in the article and was not inferred by the reviewers. Multiple articles reporting different outcomes or follow-up periods from the same parent trial are identified in the final column and should not be interpreted as independent study populations.
